# Hospital of Instruction at Algiers

**Published:** 1833-05

**Authors:** 


### REPORTS

### FROM HOSPITALS AND MEDICAL INSTITUTIONS.

### HOSPITAL OF INSTRUCTION AT ALGIERS.

*Cases of Gunshot Wounds, by* Professor Baudeus.

From among these cases, all of which are of great interest, we select the following, in which have been remarked phenomena agreeing with the opinions of modern physiologists on the functions of the fifth pair of nerves, and with regard to the seat of memory.

*Loss of Sight and of Memory, following a Gunshot Wound in the Superciliary Region.*

A captain of the 30th regiment was struck by a ball at the union of the internal third with the two external thirds of the superciliary arch of the right side. The projectile, after having splintered the external layer of the frontal sinus, lodged in the internal layer so as to compress the anterior lobe of the brain. The ball was extracted with some difficulty by means of the lever. The wounded man was carried to Algiers in a comatose state. Traumatic fever was attacked with antiphlogistics. An aërial fistula, with emphysema of the eyelid, established itself, which disappeared by the employment of nitrate of silver and a compress. The globe of the eye did not appear at all altered in structure, but its functions were completely abolished: this could be explained by the lesion of the frontal nerve of the fifth pair, the communications of this branch with the nasal branch of the same nerve, and the twigs which unite this last with the ciliary nerves of the ophthalmic ganglion. Memory was altered so that this officer lost the remembrance of his actions; objects which the evening previous had most interested him, were completely forgotten within the twenty-four hours. Frequently he was obliged to make use of the word *thing* (chose) instead of the proper expression, which escaped him in spite of all his efforts.—[From the *Revue Médicale*, February 1833.]

